# A Novel Bifunctional Self‐Stabilized Strategy Enabling 4.6 V LiCoO_2_ with Excellent Long‐Term Cyclability and High‐Rate Capability

**DOI:** 10.1002/advs.201900355

**Published:** 2019-04-24

**Authors:** Longlong Wang, Jun Ma, Chen Wang, Xinrun Yu, Ru Liu, Feng Jiang, Xingwei Sun, Aobing Du, Xinhong Zhou, Guanglei Cui

**Affiliations:** ^1^ Qingdao Industrial Energy Storage Research Institute Qingdao Institute of Bioenergy and Bioprocess Technology Chinese Academy of Sciences Qingdao 266101 P. R. China; ^2^ Center of Materials Science and Optoelectronics Engineering University of Chinese Academy of Sciences Beijing 100049 P. R. China; ^3^ College of Materials Science and Engineering Qingdao University Qingdao 266071 P. R. China; ^4^ College of Chemistry and Molecular Engineering Qingdao University of Science & Technology Qingdao 266042 P. R. China

**Keywords:** energy storage, high energy density, high voltage, LiCoO_2_ cathode, structure/interface stability

## Abstract

Although the theoretical specific capacity of LiCoO_2_ is as high as 274 mAh g^−1^, the superior electrochemical performances of LiCoO_2_ can be barely achieved due to the issues of severe structure destruction and LiCoO_2_/electrolyte interface side reactions when the upper cutoff voltage exceeds 4.5 V. Here, a bifunctional self‐stabilized strategy involving Al+Ti bulk codoping and gradient surface Mg doping is first proposed to synchronously enhance the high‐voltage (4.6 V) performances of LiCoO_2_. The comodified LiCoO_2_ (CMLCO) shows an initial discharge capacity of 224.9 mAh g^−1^ and 78% capacity retention after 200 cycles between 3.0 and 4.6 V. Excitingly, the CMLCO also exhibits a specific capacity of up to 142 mAh g^−1^ even at 10 C. Moreover, the long‐term cyclability of CMLCO/mesocarbon microbeads full cells is also enhanced significantly even at high temperature of 60 °C. The synergistic effects of this bifunctional self‐stabilized strategy on structural reversibility and interfacial stability are demonstrated by investigating the phase transitions and interface characteristics of cycled LiCoO_2_. This work will be a milestone breakthrough in the development of high‐voltage LiCoO_2_. It will also present an instructive contribution for resolving the big structural and interfacial challenges in other high‐energy‐density rechargeable batteries.

## 1 Introduction

High energy density is a pervasive and persistent concern in rechargeable batteries (e.g., Li‐, Na‐, K‐, Mg‐, Ca‐, and Zn‐ion batteries) due to the rapid upgraded requirements of portable electronics, electric vehicles, and grid energy storage.^1^ Increasing the working voltage of cathode materials is an effective strategy for higher energy density of rechargeable batteries,^2^ but it usually leads to the severe structure destruction of cathode materials and side reactions of cathode/electrolyte interfaces.^3^ Therefore, solving the big challenges from structural and interfacial instability of high‐voltage cathode materials is critical to develop high‐energy‐density rechargeable batteries.^4^

Layered α‐NaFeO_2_ structure LiCoO_2_ (LCO) was first recognized as a viable cathode material for lithium‐ion batteries (LIBs) by Goodenough and co‐workers^5^ in 1980 and successfully applied to commercial LIBs by the Sony Corporation in 1991. Since then, LCO has become the best cathode material in portable electronics for the merits of easy synthesis, high initial Coulombic efficiency, excellent cycle stability, stable charge/discharge voltage, and especially the high‐voltage plateau and high volumetric energy density. In the foreseeable future, LCO remains the best choice in the field of high‐end portable electronics benefiting from the escalated upper cutoff voltage from 4.2 to 4.45 V versus Li/Li^+^ (similarly hereinafter) in the past 28 years. Nevertheless, the strong demands for the quick renewal of the performances of consumer electronics (e.g., smartphones, iPads, notebooks) every so often have resulted in smarter, larger screened, more lightweight devices with longer standby times, which means that the LCO cathode must pierce the top limit capacity and cycle several hundred times steadily. Additionally, the fierce market competition from other cathode materials (e.g., Ni‐rich and Li‐rich layered oxides) also demands LCO to break the upper voltage limit of 4.45 V and achieve much higher energy density.[qv: 1b,6] However, ≥4.5 V LCO is greatly challenged by structure collapse and detrimental cathode/electrolyte interface reactions. On the one hand, LCO undergoes gradual phase transitions from H1 to H2, M1, H3 (>4.2 V), M2 (near 4.55 V) and O1 phases upon Li ions extraction. The phase transitions cause large anisotropic expansion and contraction along the *c* and *a* axes, respectively, above 4.2 V and oxygen layer rearrangement above 4.5 V,[qv: 1b] resulting in nonuniform stress and mechanical fractures in particles, and thus irreversible structure transition of LCO.^7^ Therefore, 4.5 V is the breakthrough point to reach much higher energy density of LCO. On the other hand, the capacity retention and safety ≥4.5 V are greatly hindered because the severe side reactions between the heavily delithiated LCO and conventional carbonate‐based electrolytes will bring about electrolyte decomposition and high‐impedance interface layers. Consequently, synchronously enhancing the structural and interfacial stability at high voltages is the key to be much closer to the capacity limit of LCO.

Substantial efforts have been made to improve the structural and interfacial stability of ≥4.5 V LCO, such as doping,^8^ coating,^9^ morphology control,^10^ and the corresponding modifications of electrolytes,^11^ separators,^12^ and binders.^13^ Doping is the most widely used strategy because it can adjust the basic physicochemical properties of materials by altering the crystal lattice at the atomic scale, such as the bandgap, defect concentration, cation ordering, and charge redistribution. Although many elements have been used as dopants for enhancing the performances of LCO at high voltages and have also been proven to be effective,[qv: 1b] but not all of elements are suitable for commercial high‐voltage LCO (HVLCO). From a commercialization perspective, cost and performance should be the two main considerations for manufacturers. Therefore, cheap and abundant elements (e.g., Si, Al, Fe, Ca, Mg, Ti, Mn) might be the first choice for use in HVLCO, while expensive elements (e.g., Ga, Bi, Rh, Zr, Ru) might be reserved only for use in fundamental research. Based on the consideration of energy density, elements (e.g., Sn, Sr, Ba, Ga, Bi, Cu, Zn, Ni, Rh, Zr) with a larger atomic mass than Co are not suitable for substituting for Co because doping with these elements would decrease the energy density of HVLCO. Additionally, to meet the needs of green development, toxic elements (e.g., Ba, B, Bi, Cu, Cr, Ni) are not recommended to be used in doped LCO.

Al doping has been confirmed to significantly enhance the structural stability of LCO host due to its same valence state, very similar ionic radius (*r*_Al_^3+^ = 0.535 Å) to those of Co (*r*_Co_^3+^ = 0.545 Å), and the much stronger Al—O bond than Co—O bond.^14^ However, the single Al doping strategy cannot evidently improve the performances of LCO at high voltages. The best performance of Al‐doped LCO reported by Zou et al.^15^ only exhibited a low initial discharge capacity of 155 mAh g^−1^ and 89% capacity retention at 0.2 C between 3.5 and 4.5 V after 50 cycles. On the other hand, quadrivalent Ti element has been reported to enhance the electronic and ionic conductivity due to charge compensation effect, the larger ionic radius (*r*_Ti_^4+^ = 0.605 Å) than Co and the formation of Li_2_TiO_3_ fast Li‐ion conductors.^16^ However, Ti‐doped LCO still suffered from poor cyclability even the high cutoff voltage was set only at 4.5 V.^15^ Therefore, Al+Ti codoping may improve the structural stability and conductivity of LCO synergistically. Additionally, Mg elements have been confirmed as the most effective dopants to increase the electronic conductivity of LCO,^17^ which can ameliorate the deficiency of insulating state for Li*_x_*CoO_2_ with high Li concentrations (*x* > 0.95).^18^ However, conventionally bulk doping LCO with Mg elements still suffers from low discharge capacity or poor cyclability even at the high cutoff voltage of 4.5 V, because a low amount of Mg^2+^ would exhibit the poor cycling stability, whereas a high amount of Mg^2+^ showed decreased specific capacity and deteriorated the energy density.^19^ Moreover, doping is incapable of modifying the superficial structure of LCO to restrain electrolyte decomposition and HF corrosion at high voltages. To the best of our knowledge, the good electrochemical performances of LCO can be barely achieved by doping alone when the upper cutoff voltage exceeds 4.5 V.[qv: 1b,20] Thereupon, gradient surface doping might be an effective strategy to deal with this dilemma, which has been demonstrated to enhance the electrochemical performances of Li‐rich or Ni‐rich cathodes due to the stabilized interfacial structure as well as the improved electronic and ionic conductivity of interfaces.^21^ Consequently, gradient surface Mg doping might be a good choice to enhance the interfacial properties of LCO at high voltages.

Herein, we proposed a novel bifunctional self‐stabilized strategy involving Al+Ti bulk codoping and gradient surface Mg doping to synchronously enhance the high‐voltage (4.6 V) structural and interfacial stability of LCO. Considering that recent modification strategies (e.g., Al+La codoping,[qv: 8c] superficial P doping,[qv: 9a] Al_2_O_3_ coating,^22^ LiAlO_2_ coating,[qv: 20a] Co_3_O_4_ coating,^23^ Li‐Al‐F‐based subsurface doping, and surface coating[qv: 20c]) only focused on improving bulk or interface structure, this bifunctional self‐stabilized strategy might lead to better performances even at the high voltage of 4.6 V. To evaluate the effects of this bifunctional self‐stabilized strategy, both the half‐cell and full‐cell performances were tested in this work. Moreover, the improved mechanism was thoroughly studied by various analysis techniques including scanning electron microscope (SEM), high resolution transmission electron microscope (HRTEM), cyclic voltammograms (CVs), in situ and ex situ X‐ray diffraction (XRD), electrochemical impedance spectroscopy (EIS), and X‐ray photoelectron spectroscopy (XPS). Our findings suggest that this bifunctional self‐stabilized strategy enormously boosts the superiority of LCO in the field of high‐end portable electronics and also presents an instructive contribution for resolving the big challenges from structural and interfacial instability of other cathode materials charged toward higher voltages in rechargeable batteries.

## 2 Results and Discussion

### 2.1 Crystal and Interfacial Structure of CMLCO

**Figure 1:**
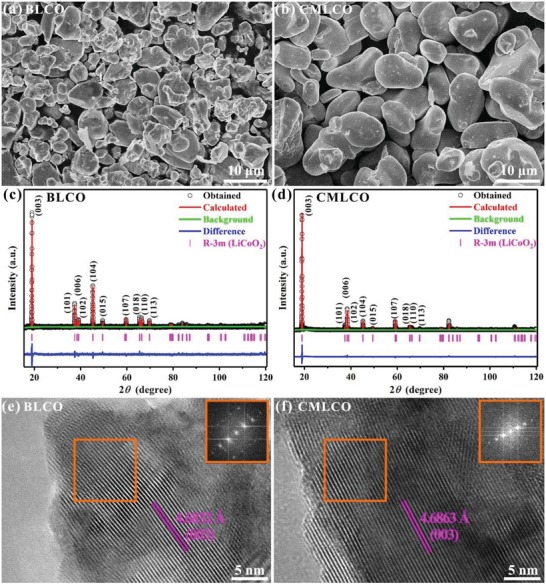
a,b) The typical SEM images of BLCO and CMLCO powders. c,d) Rietveld‐refined XRD patterns of BLCO and CMLCO powders. e,f) HRTEM images and corresponding fast Fourier transformation (FFT) calculated from the respective orange rectangular regions of BLCO and CMLCO powders.

The crystal and interfacial structure of the bare LCO (BLCO) and the comodified LCO (CMLCO) powders are characterized first. SEM images showed that both BLCO and CMLCO powders are made up of micrometer particles (**Figure** **1**a,b). The obvious difference is that CMLCO powders possess much more and larger particles with smooth surfaces. The XRD results (Figure 1c,d and **Table** **1**) indicated that both BLCO and CMLCO were generally corresponded to hexagonal *R‐*3*m* structure. Clear peak separation of (006)/(102) and (108)/(110) in BLCO and CMLCO indicates that both as‐prepared samples have a well‐developed crystalline layered structure.^24^ Additionally, the peak intensity ratio of (003)/(104) in BLCO (1.956) and CMLCO (6.795) is both larger than 1.2, which indicates that the degree of cationic disorder in as‐prepared samples is lower.^25^ By contrast, CMLCO possesses a more ordered layered structure than BLCO due to the larger peak intensity ratio of (003)/(104). The chemical composition ratio of Al:Ti:Mg:Co was measured by inductively coupled plasma optical emission spectrometry (ICP‐OES) and estimated to be 3:1:8:1000. The refinement results of CMLCO are very good when Al and Ti occupy 3*b* sites^26^ and Mg ions occupy 3*a* sites.[qv: 9b,27] Moreover, a slight increase in lattice parameters is found after comodifying, which seems to be associated with the pillaring effect of Ti^4+^ ions. The HRTEM images (Figure 1e,f) show that both BLCO and CMLCO exhibit clear lattice stripes and diffraction patterns of (003), indicating that the as‐prepared samples exhibit well‐order layered structures. However, after comodifying, the interplanar spacing of (003) is slightly larger, which is consistent with the Rietveld‐refined XRD results.

**Table 1:** Refined structure parameters of BLCO and CMLCO

Atom	Site	*x*	*y*	*z*	Occupancy	*U*_iso_
BLCO (*R*_wp_ = 1.31%, *R*_p_ = 0.86%, χ^2^ = 1.231) LiCoO_2_ (Space group: *R‐*3*m*) Lattice parameters: *a* = *b* = 2.8158(5) Å, *c* = 14.0513(2) Å, α = β = 90°, γ = 120°, Volume = 96.4869 Å^3^						
Li	3*a*	0.00000	0.00000	0.00000	1.000	0.014(6)
Co	3*b*	0.00000	0.00000	0.50000	1.000	0.023(8)
O	6*c*	0.00000	0.00000	0.2300(6)	1.000	0.049(1)
CMLCO (*R*_wp_ = 3.37%, *R*_p_ = 1.70%, χ^2^ = 1.023) LiCoO_2_ (Space group: *R‐*3*m*) Lattice parameters: *a* = *b* = 2.8166(3) Å, *c* = 14.0560(3) Å, α = β = 90°, γ = 120°, Volume = 96.5720 Å^3^						
Li	3*a*	0.00000	0.00000	0.00000	0.98(1)	0.020(1)
Mg	3*a*	0.00000	0.00000	0.00000	0.01(9)	0.020(1)
Co	3*b*	0.00000	0.00000	0.50000	0.99(7)	0.001(2)
Al	3*b*	0.00000	0.00000	0.50000	0.002(0)	0.001(2)
Ti	3*b*	0.00000	0.00000	0.50000	0.001(0)	0.001(2)
O	6*c*	0.00000	0.00000	0.2476(3)	1.000	0.068(5)

**Figure 2:**
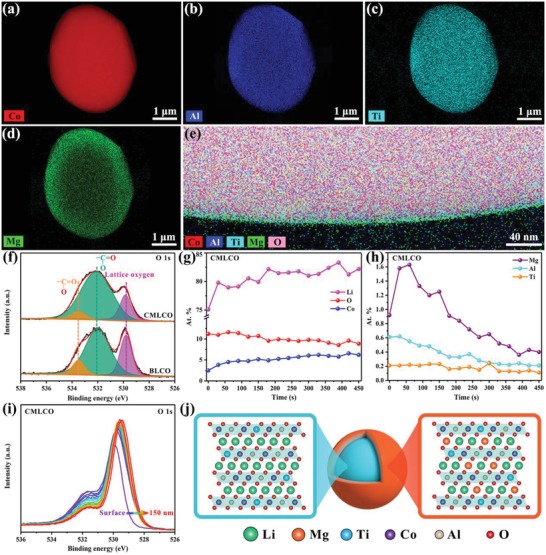
a–e) HAADF‐STEM mappings of Co, Al, Ti, and Mg elements and superposed distribution of Co, Al, Ti, Mg, and O elements in CMLCO powders. f) O 1s XPS spectra of BLCO and CMLCO powders. g–i) The Li, O, Co, Mg, Al, Ti element contents and corresponding O 1s XPS spectra in the surface of CMLCO obtained from XPS depth profiles with increasing of Ar^+^ etching time up to 450 s. j) Schematic illustration of the structure design of CMLCO with Al+Ti bulk codoping (blue core) and gradient surface Mg doping (orange shell).

To further figure out the distribution of comodified elements, high‐angle annular‐dark‐field scanning transmission electron microscope (HAADF‐STEM) mapping was conducted for CMLCO powders (**Figure** **2**a–d) due to the very limited doping contents (<0.4 wt%). It can be found that the Co, Al, and Ti elements distribute uniformly within the CMLCO particles, while the Mg elements enrich on the surface of CMLCO particles with gradient distribution. This phenomenon can be seen more clearly in the superposed distribution of Co, Al, Ti, Mg, and O elements as shown in Figure 2e. It can be inferred that the surface layer on CMLCO is the multielement doped Li_1−_ *_x_*Mg*_x_*Co_1−_ *_y_*_−_ *_z_*Al*_y_*Ti*_z_*O_2+_ *_δ_* with Mg gradient distribution. The surfacial and subsurfacial element distribution and corresponding valence variation after comodifying were also studied via XPS due to its high surface sensitivity.^28^ As shown in Figure 2f, the O 1s spectra of BLCO and CMLCO powders consist of three components,^29^ that is lattice oxygen in the O3 layered LCO structure (≈529.8 eV), oxygen atoms doubly bound to carbon atoms (532.0 eV) and oxygen bound to carbon with a single bond (533.5 eV). It can be found that there is basically no change in the amount of Li_2_CO_3_ (532.0 and 533.5 eV) after comodifying, which indicates that this bifunctional self‐stabilized strategy does not change the air stability of LCO.[qv: 24b] The variation after comodifying is that the binding energy of lattice oxygen increased 0.1 eV, which might be due to the appearance of Al—O and Ti—O with higher bond energy than Co—O.[qv: 1b] Moreover, divalent Mg ions occupied the 3a sites of Li on the surface of CMLCO, which also increased the attraction to electron clouds near the lattice oxygen and thus resulted in slightly higher binding energy. To further confirm the element distribution, the XPS depth profiles (Figure 2g,h) were conducted by increasing of Ar^+^ etching time up to 450 s (≈150 nm). It can be found that the variation of O, Co, Al, and Ti element contents is relatively small, while the variation of Li and Mg element contents is slightly large. Due to more Mg ions occupying the 3a sites of Li on the surface, the Li element content on the surface is slightly lower than that in the bulk. Additionally, with the Mg element content decreasing toward the bulk, the binding energy of lattice oxygen gradually declined due to the reduced attraction to electron clouds near the lattice oxygen (Figure 2i). Therefore, it can be concluded that we have successfully synthesized the CMLCO with Al+Ti bulk codoping and gradient surface Mg doping (Figure 2j).

### 2.2 Electrochemical Performances of CMLCO

**Figure 3:**
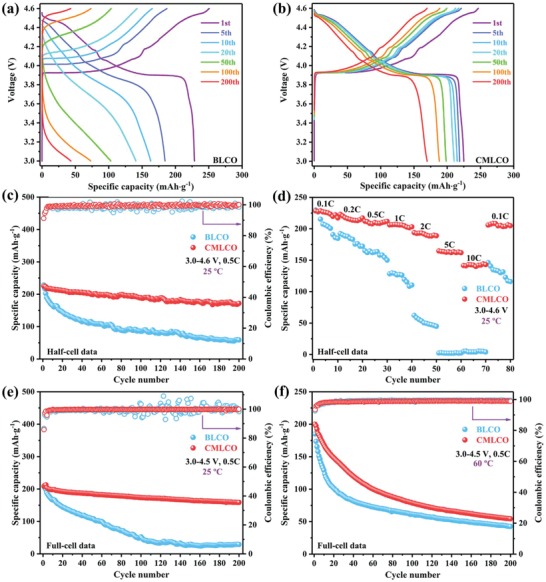
a,b) The galvanostatic charge–discharge curves of BLCO and CMLCO in half‐cell configuration at different cycles. c,d) The cycling performance and rate capability of BLCO and CMLCO in half‐cell configuration. e,f) The cycling performance of BLCO and CMLCO in full‐cell configuration at 25 and 60 °C.

In order to study the effects of the bifunctional self‐stabilized strategy, the electrochemical performances of BLCO and CMLCO in half‐cell (3.0–4.6 V) and full‐cell (3.0–4.5 V) configuration were tested. As shown in **Figure** **3**a, BLCO cathode exhibited a discharge capacity of 228.5 mAh g^−1^ at the current density of 0.1 C in the initial cycle, but suffered from rapid capacity decay (from 228.5 to 43.3 mAh g^−1^) due to the increased overpotential and voltage drop in the initial state of discharging during cycling. By sharp contrast, the CMLCO cathode showed high initial discharge capacity (224.9 mAh g^−1^) and slight overpotential. Even more remarkably, the comodified samples still exhibited a discharge capacity of 169.9 mAh g^−1^ at the 200th cycle, which was nearly four times that of BLCO (Figure 3b). As a result, the CMLCO cathode showed 78% capacity retention after 200 cycles at current density of 0.5 C, whereas the BLCO cathode only displayed 23% capacity retention (Figure 3c). In Figure 3d, the CMLCO cathode could exhibit a specific capacity of up to 142 mAh g^−1^ even at the high current density of 10 C (1400 mA g^−1^), whereas the discharge capacity of BLCO cathode descended to zero at 5 C and its cyclic degradation was obvious even at 0.1 C. The significantly improved rate capability after comodifying can be attributed to the higher Li‐ion diffusivities in CMLCO as discussed below. Studies on LCO‐based full cells are highly necessary before they access the markets for commercial applications, thus the full‐cell performances are also tested (Figure 3e; Figure S1, Supporting Information). The CMLCO/mesocarbon microbeads (MCMB) full cell showed an initial discharge capacity of 208.2 mAh g^−1^ and exhibited 78% capacity retention after 200 cycles at 0.5 C. The BLCO/MCMB full cells displayed a similar initial discharge capacity (210.2 mAh g^−1^) but very poor cycling performance (13.7% capacity retention after 200 cycles). More importantly, the excellent performances of CMLCO‐based half and full cells are also better than that of previously reported LCO‐based cells in view of discharge capacity and cyclability both at the high cutoff voltage of 4.5 V (Figure S2 and Table S1, Supporting Information) and 4.6 V (Table S2, Supporting Information), which confirms the advanced effect of this bifunctional self‐stabilized strategy. Moreover, we also first reported the high‐temperature performance (60 °C) of LCO‐based full cells. As shown in Figure 3f, both CMLCO/MCMB and BLCO/MCMB cells suffered from rapid capacity decay at the harsh conditions of high temperature and high cutoff voltage, but the CMLCO/MCMB batteries still exhibited better cyclability due to the bifunctional self‐stabilized strategy for LCO cathode (Figure S3, Supporting Information). It should be noted that the high‐temperature performance of CMLCO/MCMB can be further improved by the optimal design of electrolytes, binders, separators, and/or the ratio of cathode and anode, which is the next work for our group. The preliminary studies of CMLCO/MCMB full cells at 60 °C further confirm the role of our strategy and indicate the reported CMLCO is a promising cathode material for commercial applications in high‐end portable electronics.

### 2.3 Structural Reversibility of CMLCO

**Figure 4:**
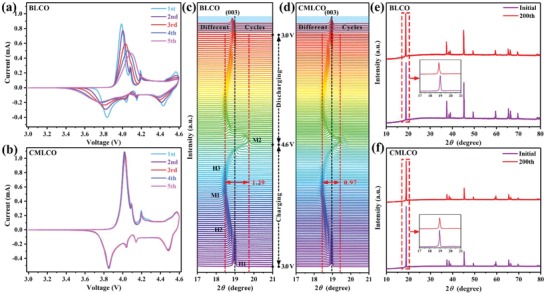
a,b) The initial five CVs of BLCO and CMLCO in half‐cell configuration. c,d) The (003) peaks evolution of in situ XRD characterization for BLCO and CMLCO cathodes during the first charge–discharge process, and ex situ XRD characterization for BLCO and CMLCO cathodes after 5th, 10th, 20th, 5th, 100th, and 200th cycles. e,f) The XRD patterns of BLCO and CMLCO cathodes before cycling and after 200 cycles.

To illuminate the reasons for the improved electrochemical performances after comodifying, the structural transition of BLCO and CMLCO cathode was first studied. **Figure** **4**a,b displays the initial five CV profiles carried out between 3.0 and 4.6 V. It can be observed that both BLCO and CMLCO cathodes exhibited similar redox peaks (or phase‐transition peaks) in the first CV profile (≈3.92 V, H1/H2; ≈4.09 V, H2/M1; ≈4.16 V, M1/H3; ≈4.55 V, H3/M2), but with the cycle number increasing, the phase transitions of H2/M1 (≈4.09 V), M1/H3 (≈4.16 V), and H3/M2 (≈4.55 V) gradually weakened or disappeared. Moreover, the potential differences between the anodic and cathodic peaks in the CV curves also increased from 0.15 to 0.35 V, which indicated that the BLCO cathode suffered from larger polarization. On the other hand, as we expected, the CMLCO cathode showed excellent reversible redox peaks and very small polarization, which suggests high structural reversibility and might contribute to the outstanding electrochemical performances as shown in Figure 3.

For the sake of giving the accurate structure variations during charging and discharging, in situ and ex situ XRD were also tested (Figure 4c–f; Figures S4–S6, Supporting Information). In the XRD spectra of hexagonal layered structure LCO, the position changes of (003) peak represent the variation of *c* value, while (101) represents the variation of *a* and *b* values.^30^ Amatucci et al.^31^ have found that during Li ions removal from LCO, the lattice parameter *c* value changed significantly, while the *a* and *b* values changed only slightly. Therefore, the (003) peak evolution during charging and discharging basically reflects the change of LCO volume. Hence, we mainly focused on the variation of (003) peak when analyzing in situ and ex situ XRD results. As shown in Figure 4c,d, both BLCO and CMLCO cathode experienced a similar phase transition (H1/H2; H2/M1; M1/H3; H3/M2), which is consistent with results of CVs. However, the (003) peak of BLCO exhibited a greater amplitude (1.29°) of variation than that of CMLCO (0.97°). This different evolution of (003) peak indicates that BLCO suffered from larger volume change than CMLCO during charging and discharging, which will result in the severe structure deterioration (e.g., cracks, irreversible phase transition) especially after long‐term cycles. As displayed in Figure S7 (Supporting Information), the BLCO cathode emerged large cracks after 200 cycles while the CMLCO cathode retained good integrity. On the other hand, the (003) peak moved slightly toward the lower degree after the initial cycle (Figure 4c). Moreover, with the cycle number increasing, this shift of (003) peak is more pronounced. It means that the irreversible structure transition gradually accumulates in BLCO during 200 cycles (Figure 4c,e), which is might due to the irreversible loss of Co and O during the repeated charging and discharging.[qv: 9a] As an obvious comparison, the CMLCO exhibited better structural reversibility and integrity even after 200 cycles due to the suppressed volume change (Figure 4d,f; Figure S7b, Supporting Information). It might result from the larger van der Waals gap in the CMLCO compared to BLCO due to the much stronger Al—O bond than Co—O bond after Al bulk doping. Hence, lower cycling‐induced lattice strain is possible in CMLCO cathode. Moreover, the larger Ti ions could work as a pillar to facilitate the (de)intercalation of the Li ions in the lattice and prevent the distortion of the structure. Therefore, it can be concluded that this bifunctional self‐stabilized strategy can significantly enhance the structural stability of LCO even cycled between 3.0 and 4.6 V.

### 2.4 Interfacial Stability of CMLCO

**Figure 5:**
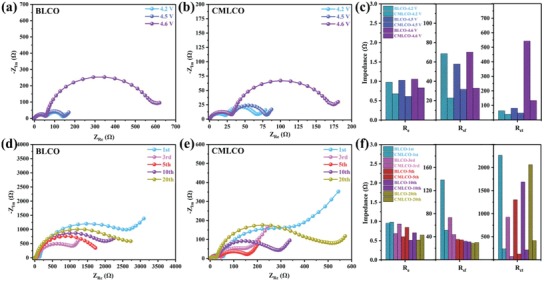
a–c) The variation in the resistances of BLCO and CMLCO charged up to 4.2, 4.5, and 4.6 V at first cycle. d–f) The variation in the resistances of BLCO and CMLCO fully discharged at 1st, 3rd, 5th, 10th, and 20th cycles.

The interfacial stability is also closely related to the improved electrochemical properties after comodifying. Therefore, we then studied the interfacial impedances at different high cutoff voltages and cycle numbers by means of EIS. The equivalent circuit used for fitting the experimental EIS data is shown in Figure S8 (Supporting Information). As shown in **Figure** **5**a–c and Table S3 (Supporting Information), when the high cutoff voltage was increased from 4.2 to 4.6 V, the electrolyte resistances (*R*_e_) in BLCO‐ and CMLCO‐based LIBs remained almost unchanged (≈1 Ω), whereas the charge‐transfer resistances (*R*_ct_) and surface film (electrolyte/electrode interface) resistances (*R*_sf_) exhibited different variations. Observably, the increase of *R*_ct_ is more prominent especially when the higher cutoff voltage is raised from 4.5 to 4.6 V, which might result from the lower Li‐ion diffusivities after high delithiation. Compared to BLCO, CMLCO exhibits lower *R*_ct_ because the Li‐ion diffusivity of CMLCO at 4.6 V (1.0390 × 10^−13^ cm^2^ g^−1^) is one order of magnitude higher than that of BLCO (1.1556 × 10^−14^ cm^2^ g^−1^) (Figure S9, Supporting Information). On the other hand, the *R*_sf_ of BLCO reduced when the upper cutoff voltage is increased from 4.2 to 4.5 V, which is related to the cathode electrolyte interfaces (CEI) layer decompositon^32^ caused by the lattice stress of LCO. However, when the upper cutoff voltage is increased from 4.5 to 4.6 V, the *R*_sf_ is further increased, which might be because the interfacial side reaction between the highly oxidized cathode and the electrolyte was more severe than the decomposition of CEI. On the contrary, the *R*_sf_ of CMLCO is lower than that of BLCO and does not decrease during charging, indicating reduced interfacial reactions and decomposition of CEI. This is because the Mg‐rich multielement doped surface layer (probably Li_1−_ *_x_*Mg*_x_*Co_1−_ *_y_*_−_ *_z_*Al*_y_*Ti*_z_*O_2+_ *_δ_*) could stabilize electrode/electrolyte interface and exhibit good electrochemical stability,[qv: 8a] enhancing electronic and ionic conductivity.[qv: 9b,26b] Resistance variations have a critical effect on the long‐term cyclability. The EIS results of BLCO after different cycles (Figure 5d,f; Figure S10a and Table S4, Supporting Information) showed that the *R*_sf_ of BLCO markedly dropped from the initial to the third cycle, and continued to decrease slowly in the following cycles, suggesting the stabilization process of CEI/SEI formation in the first three cycles. By contrast, the *R*_sf_ of CMLCO exhibited less variation once CEI formation in the first cycle (Figure 5e,f; Figure S10b and Table S4, Supporting Information). The formation of CEI film on the BLCO and CMLCO cathode after the initial cycle was also studied by the HRTEM images. As shown in Figure S11 (Supporting Information), it can be found that the CEI film on the BLCO cathode was thick and discontinuous because the exposed highly oxidized surfaces boosted the electrolyte decomposition and the larger volume change caused the fracture of the formed CEI film. As a sharp contrast, the CEI film on the CMLCO cathode was thinner and continuous due to the reduced interfacial side reaction and smaller volume change. Therefore, CMLCO can save the precycling process, which is usually necessary to completely stabilize the CEI film on the surface of BLCO.[qv: 9a] Additionally, the *R*_ct_ of CMLCO is an order of magnitude lower than that of BLCO. Therefore, the CMLCO‐based LIBs can exhibit excellent electrochemical performances and low overpotential as shown in Figure 3.

**Figure 6:**
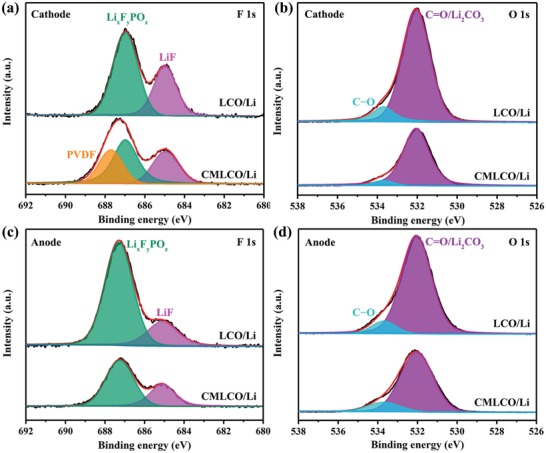
XPS spectra of a,c) F 1s and b,d) O 1s of the cathode and anode disassembled from LCO/Li and CMLCO/Li batteries after the initial cycle.

The evolution of CEI (cathode side) and SEI (anode side) after the initial cycle was studied via XPS (**Figure** **6**). The F 1s core level peaks of BLCO and CMLCO cathodes were primarily assigned to LiF (at 685 eV) and Li*_x_*F*_y_*PO*_z_* (at 687 eV).^29^ It has been well known that LiF and Li*_x_*F*_y_*PO*_z_*, the main F‐containing components in CEI or SEI in LiPF_6_‐based electrolytes, can result in a serious capacity fading during cycling especially in the high‐voltage range. The intensities of LiF and Li*_x_*F*_y_*PO*_z_* peaks in BLCO were evidently larger than that in CMLCO, which indicated that the CEI film formed on the surface of BLCO was much thicker than that on the surface of CMLCO. On the other hand, considering the limited analysis depth of XPS, the relative intensity of the polyvinylidene difluoride (PVDF) peak may serve as an indicator of CEI thickness. As shown in Figure 6a, PVDF peak (687.8 eV) did not appear in BLCO cathode while existed in CMLCO cathode, which also indicated that the thickness of CEI in BLCO cathode was larger than that in CMLCO cathode. As shown in Figure 6b, the O 1s spectra for the CEI film formed after the initial cycle, revealed the relative ratio of carbonates (C—O and C=O) in BLCO and CMLCO cathodes. It can be found that the amount of carbonate species was drastically reduced after comodifying. The presence of more carbonate species on the surface BLCO is known to lead to higher impedance growth, which is consistent with the poor cyclabiliy of BLCO as shown in Figure 3. It has been reported that the certain transition‐metal ions dissolved from the cathode would diffuse to anode side and disrupted SEI.^33^ Consequently, we also analyzed the components of SEI on the anode. As displayed in Figure 6c,d, the intensity of LiF, Li*_x_*F*_y_*PO*_z_*, and Li_2_CO_3_/C=O in BLCO/Li batteries was evidently larger than that in CMLCO/Li batteries. It is concluded that this comodifying strategy can also enhance the interfacial stability on anode side maybe due to suppressed dissolution of Co ions (Figure S12, Supporting Information).

## 3 Conclusion

In summary, we have succeeded in developing an innovative method to enhance the structural and interfacial stability of LCO charged to 4.6 V via a bifunctional self‐stabilized strategy involving Al+Ti bulk codoping and gradient surface Mg doping. This bifunctional self‐stabilized strategy also remarkably enhanced the Li‐ion diffusivity in LCO. Therefore, the CMLCO cathode could display an initial discharge capacity 224.9 mAh g^−1^ and 78% capacity retention after 200 cycles between 3.0 and 4.6 V. Excitingly, the CMLCO cathode exhibited a specific capacity of up to 142 mAh g^−1^ even at the high current density of 10 C, which is the 63% capacity retention of that at 0.1 C. Moreover, when coupled with the MCMB anode, the CMLCO‐based full cells also displayed improved capacity retention even at the high temperature of 60 °C. Nevertheless, the long‐term cyclability at high temperature should be further enhanced in the future. Furthermore, some more in‐depth analyses (including the direct evidences of element occupancy, surface composition, electronic conductivity, etc.) are also needed to elaborate the reasons of performance improvements. Our findings suggest that this bifunctional self‐stabilized strategy enormously boosts the superiority of LCO in the field of high‐end portable electronics and also presents an instructive contribution for resolving the big challenges from structural and interfacial instability of other cathode materials charged toward higher voltages in rechargeable batteries.

## 4 Experimental Section

*Material Synthesis*: Typically, CoSO_4_·7H_2_O and Al_2_(SO_4_)_3_ were first dissolved in deionized water to form a clear aqueous solution. After that, the as‐prepared solution was slowly added to a mixed solution of 2 m NaOH solution and NH_3_·H_2_O (10:1 in volume) under vigorous stirring at 55 °C while the pH of reaction solution was controled at 11.5 by NH_3_·H_2_O. After stirring for 30 min, the obtained precipitation was filtered, washed by deionized water, and finally dried at 80 °C overnight and then at 120 °C in the vacuum for 4 h to obtain Al‐doped Co(OH)_2_ precursors. Then the mixture of the as‐obtained Al‐doped Co(OH)_2_ precursors, TiO_2_ and Li_2_CO_3_ were first heated at 650 °C for 6 h, and then sintered at 1000 °C for 12 h in air atmosphere to obtain the semi‐finished products. Then the semi‐finished products, MgSO_4_, NaOH, and NH_3_·H_2_O were used to prepare Mg(OH)_2_ uniformly coated samples at a specific pH value. Finally, the CMLCO powders were synthesized by heat treatment of the coated samples at 850 °C for 2 h. The chemical composition ratio of Al:Ti:Mg:Co was measured by ICP‐OES (Agilent 730) and estimated to be 3:1:8:1000. As a comparison, BLCO was synthetized by the same procedure as CMLCO but without corresponding Al‐, Ti‐, and Mg‐based additives.

*Materials Characterization*: The high quality powder XRD patterns were collected using an X‐ray diffractometer (Rigaku SmartLab) equipped with a Cu Kα radiation source (λ_1_ = 1.54060 Å, λ_2_ = 1.54439 Å) by measuring the diffraction angle (2θ) between 10° and 120° with a scanning rate of 1 s step^−1^ and a step size of 0.01°. Rietveld refinement was then conducted using the GSAS software to obtain crystal structure parameters.^34^ For in situ XRD experiment during battery test, a specially designed Swagelok cell equipped with an aluminum window for X‐ray penetration. The in situ XRD patterns were collected at 2θ = 15°−80° every ten minutes. The HRTEM images were characterized by TEM (FEI Tecnai G^2^ F30, 300 kV). HAADF mappings were executed using a JEOL ARM200F (JEOL, Tokyo, Japan) STEM with an accelerating voltage of 200 kV with a thermal filed‐emission gun and a probe Cs corrector (CEOS GmbH, Heidelberg, Germany). The XPS measurement was performed on an ESCALab 250Xi (Thermo Scientific) spectrometer equipped with an Al Kα achromatic X‐ray source.

*Electrochemical Measurements*: In the half‐cell configuration, the electrochemical evaluation was performed in a CR2032‐type coin cell with a lithium metal anode as a counter electrode and microporous polypropylene as a separator. The BLCO and CMLCO electrodes were prepared by coating N‐methyl‐2‐pyrrolidone (NMP)‐based slurries comprising 80 wt% active materials, 10 wt% PVDF binder, and 10 wt% super P on aluminum current collectors. After drying and pressing, the electrodes were punched into disks with a diameter of 12 mm. The average active material loading densities of BLCO and CMLCO electrodes were corresponded to 1.5 ± 0.2 mg cm^−2^. In the full‐cell configuration, the cathode electrodes were consisted of 96 wt% active materials, 2 wt% Super P, and 2 wt% PVDF binder. The anode electrodes were consisted of 93 wt% MCMB, 2 wt% Super P, and 5 wt% aqueous binder (LA133). The slurries for cathode (NMP as solvent) and anode (H_2_O as solvent) were coated on aluminum foil and copper foil, respectively. After drying and pressing, the cathode and anode electrodes were punched into disks with different diameters (12 mm for cathode electrodes and 14 mm for anode electrodes). The electrode loading levels of cathode and anode were 7 ± 0.2 and 5 ± 0.2 mg cm^−2^, respectively. All punched electrodes were dried at 120 °C for 24 h prior to the assembling operation in an Ar‐filled glovebox. The half cells were tested between 3.0 and 4.6 V in the commercialized electrolyte solution of 1.15 m LiPF_6_ in ethylene carbonate/dimethyl carbonate/diethylene carbonate ( = 3:4:3 vol%). The full cells (1.05 ± 0.1 N/P capacity ratio) were tested between 3.0 and 4.5 V. Galvanostatic cycling tests were conducted using Land battery test system (Land CT2001A, Wuhan Land Electronic Co. Ltd., China). The CV and EIS tests were carried out by an electrochemical working station (Biologic VMP‐300). In half‐cell configuration, the CV tests were carried out at a scan rate of 0.1 mV s^−1^ under 3.0–4.6 V. The EIS tests were performed over a frequency range of 100 kHz to 5 mHz with an applied amplitude of 5 mV.

## Conflict of Interest

The authors declare no conflict of interest.

## Supporting information

Supplementary
