# Supplementary material for: A Novel Bifunctional Self‐Stabilized Strategy Enabling 4.6 V LiCoO2 with Excellent Long‐Term Cyclability and High‐Rate Capability
Source: Adv Sci (Weinh). 2019 Apr 24;6(12):1900355. doi: 10.1002/advs.201900355 (PMC6662074; doi:10.1002/advs.201900355)
Supplement: Supplementary file 1 — Supplementary [file ADVS-6-1900355-s001.pdf]

Copyright WILEY-VCH Verlag GmbH & Co. KGaA, 69469 Weinheim, Germany, 2019.

Supporting Information

for *Adv. Sci.,* DOI: 10.1002/advs.201900355

A Novel Bifunctional Self-Stabilized Strategy Enabling 4.6 V $LiCoO_2$ with Excellent Long-Term Cyclability and High-Rate Capability

Longlong Wang, Jun Ma, Chen Wang, Xinrun Yu, Ru Liu, Feng Jiang, Xingwei Sun, Aobing Du, Xinhong Zhou, and Guanglei Cui**

Copyright WILEY-VCH Verlag GmbH & Co. KGaA, 69469 Weinheim, Germany, 2019.

Supporting Information

A Novel Bifunctional Self-stabilized Strategy Enabling 4.6 V $LiCoO_2$ with Excellent Long-term Cyclability and High-rate Capability

Longlong Wang, Jun Ma, Chen Wang, Xinrun Yu, Ru Liu, Feng Jiang, Xingwei Sun, Aobing Du, Xinhong Zhou and Guanglei Cui**

L. Wang, J. Ma, C. Wang, R. Liu, A. Du, Prof. G. Cui
Qingdao Industrial Energy Storage Research Institute, Qingdao Institute of Bioenergy and Bioprocess Technology, Chinese Academy of Sciences, Qingdao, 266101, P. R. China.
E-mail: majun@qibebt.ac.cn, cuigl@qibebt.ac.cn

L. Wang, C. Wang, A. Du
Center of Materials Science and Optoelectronics Engineering, University of Chinese Academy of Sciences, Beijing 100049, P. R. China

X. Yu
Qingdao University, Qingdao, 266071, PR China

F. Jiang, X. Sun, Prof. X. Zhou
College of Chemistry and Molecular Engineering, Qingdao University of Science & Technology, Qingdao 266042, P. R. China

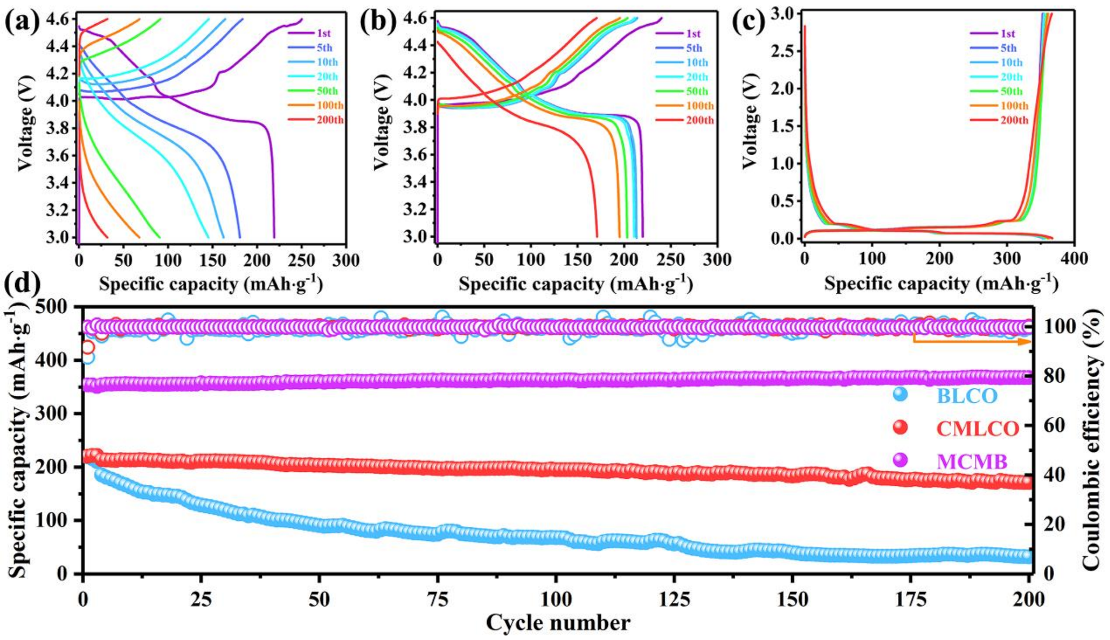

Figure S1 The half cells galvanostatic charge–discharge curves at different cycles and cycling performance of BLCO (3.0-4.6 V, 0.5 C), CMLCO (3.0-4.6 V, 0.5 C) and MCMB (0.005-3 V, 0.5 C) electrodes used for full cells at 25 °C.

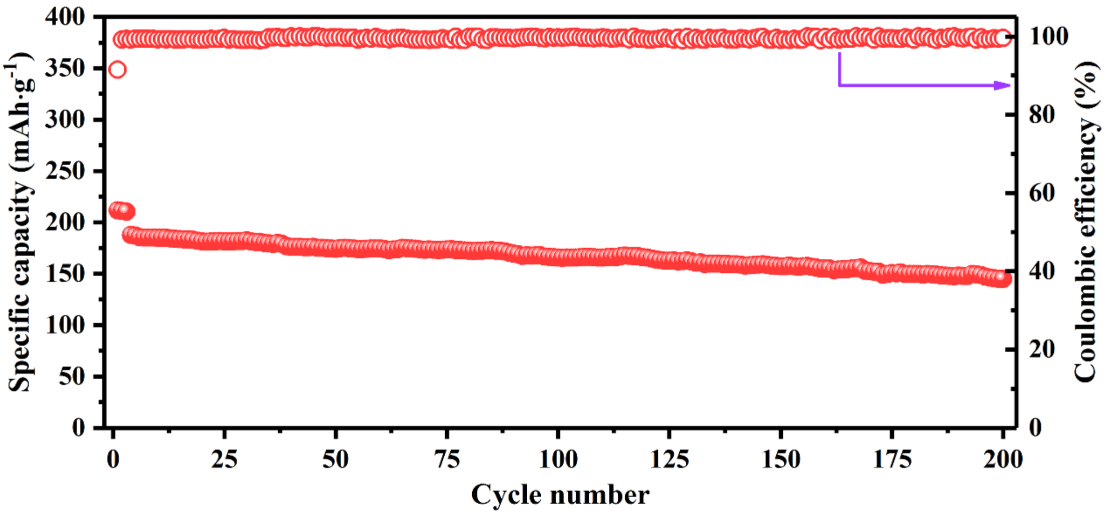

Figure S2 The cycling performance of CMLCO/Li half cells between 3.0 and 4.5 V at current density of 1 C at 25 °C.

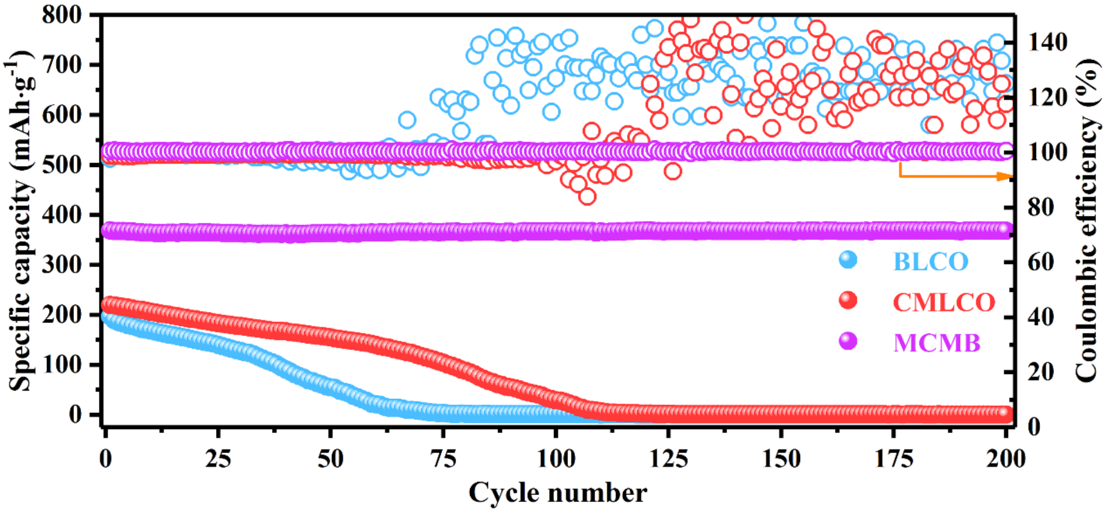

Figure S3 The half cells cycling performance of BLCO (3.0-4.6 V, 0.5 C), CMLCO (3.0-4.6 V, 0.5 C) and MCMB (0.005-3 V, 0.5 C) electrodes used for full cells at 60 °C.

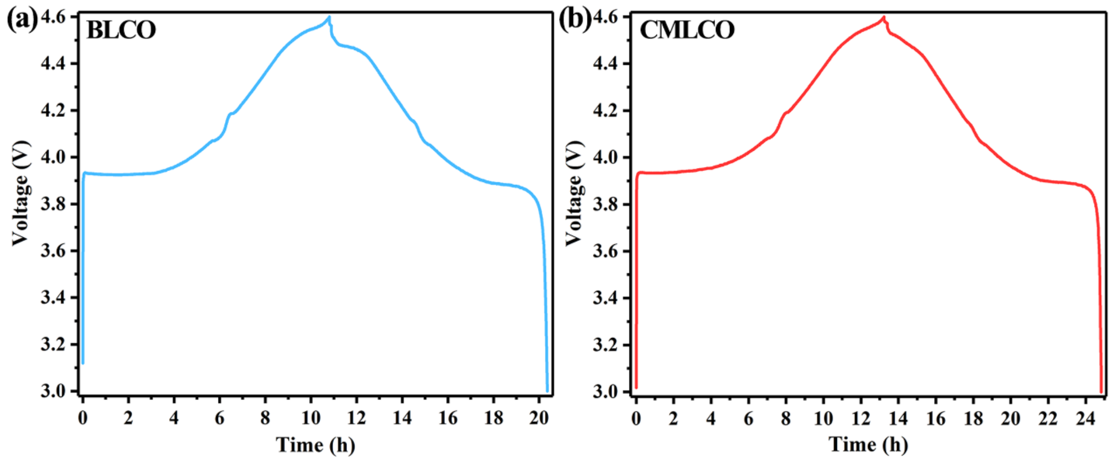

Figure S4 The galvanostatic charge–discharge curves of BLCO and CMLCO cathode during in-situ XRD characterization.

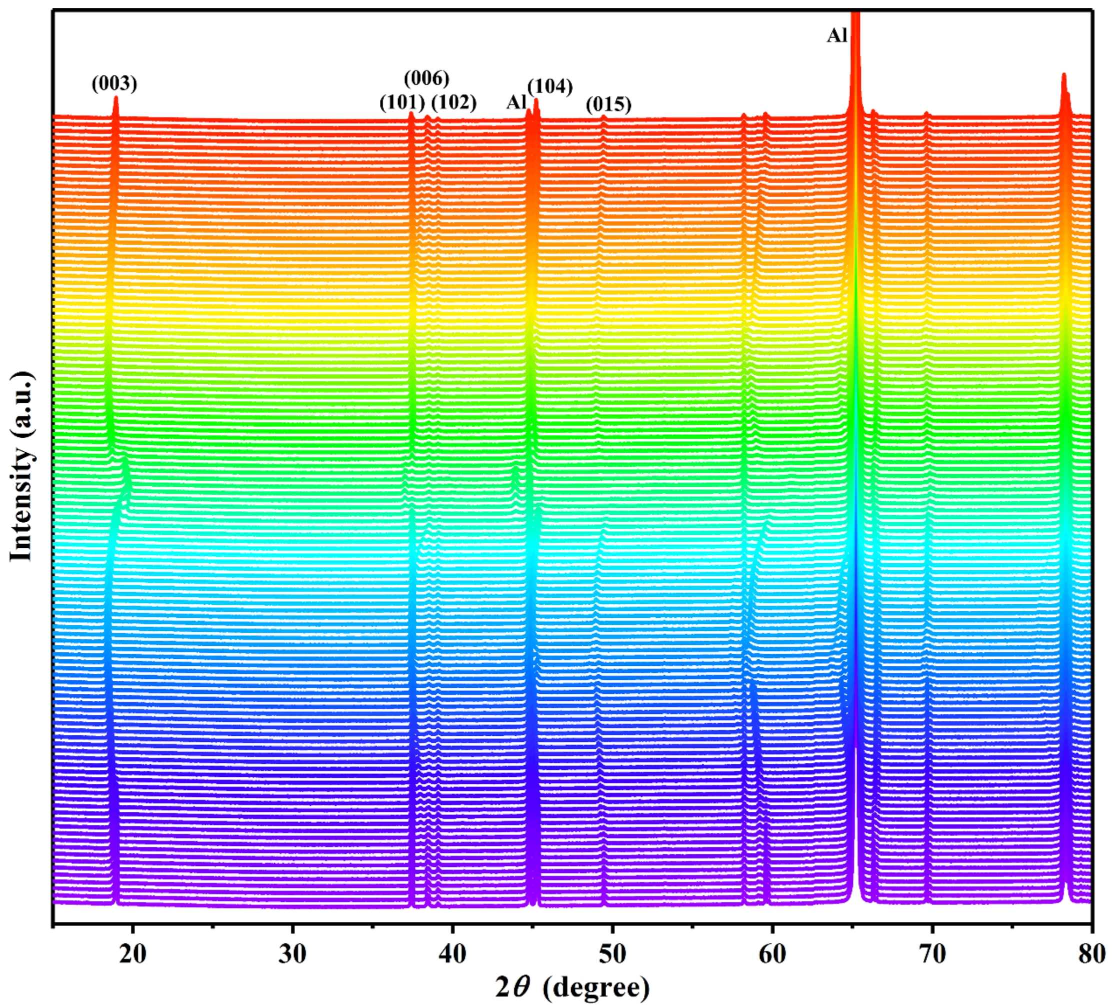

Figure S5 The full spectrums of *in-situ* XRD characterization for BLCO cathode during the first charge–discharge process.

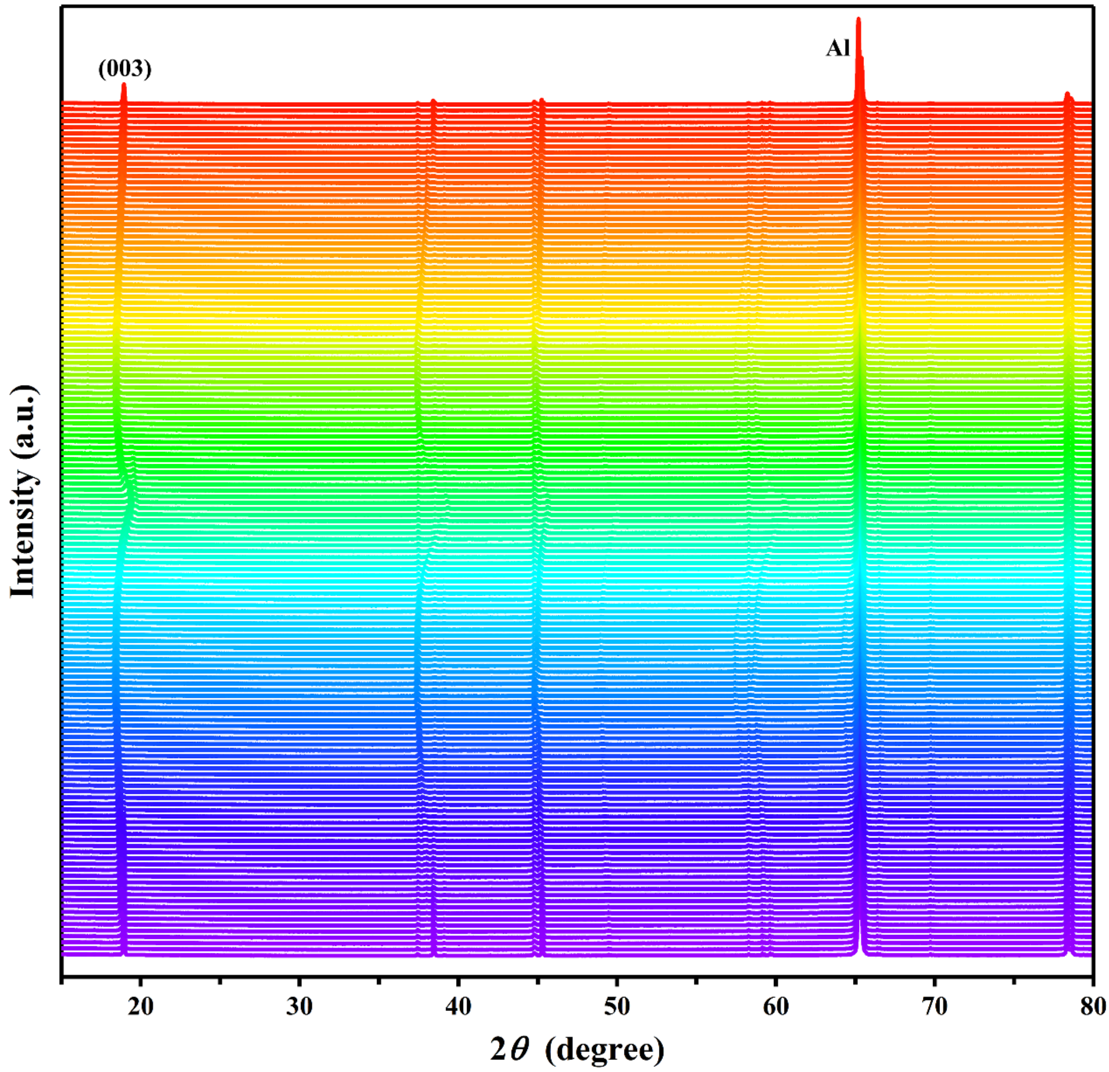

Figure S6 The full spectrums of *in-situ* XRD characterization for CMLCO cathode during the first charge–discharge process.

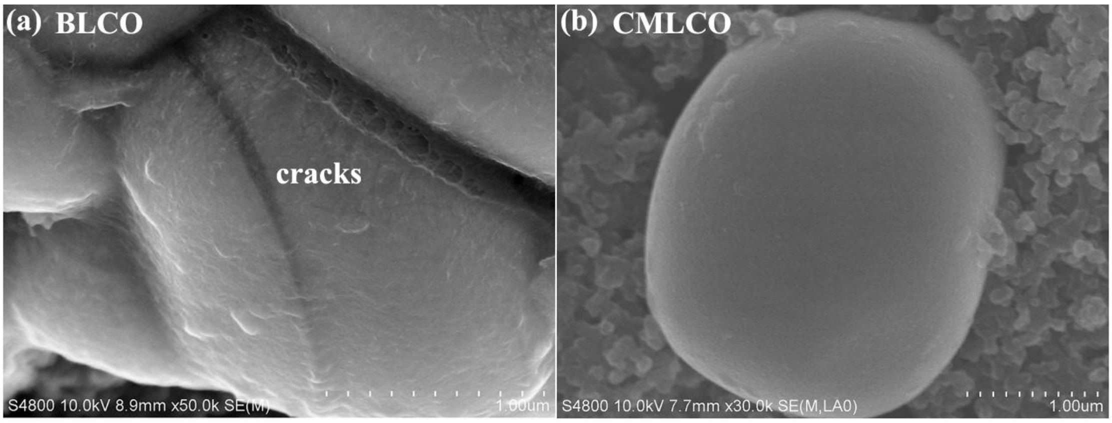

Figure S7 The typical SEM images of BLCO and CMLCO cathodes cycled between 3.0 and 4.6 V after 200 cycles at 25 °C.

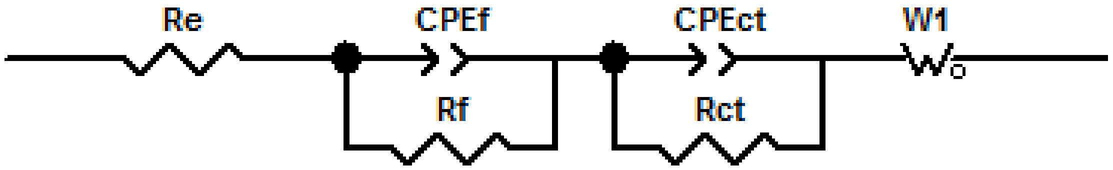

Figure S8 The equivalent circuit used for fitting the experimental EIS data.

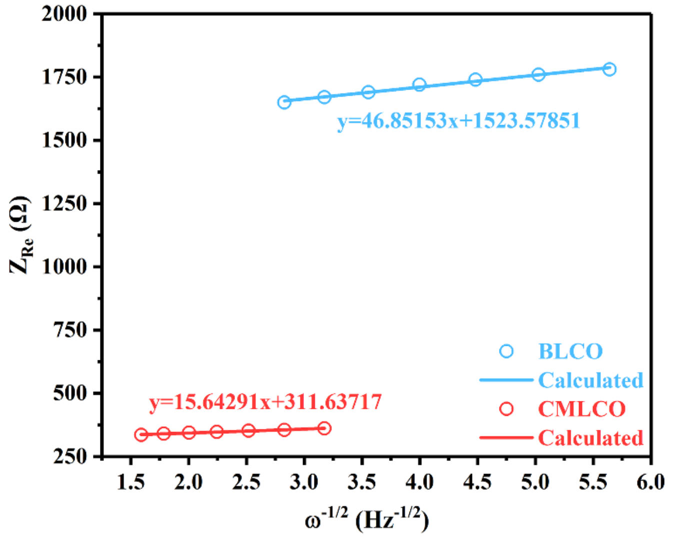

Figure S9 The relationship between Z_{Re} and the square root of frequency ($\omega^{-1/2}$) in the low frequency region plots of BLCO and CMLCO cathode with the charge cutoff voltages of 4.6 V.

The Li ion diffusivities (D) of BLCO and CMLCO at 4.6 V were calculated using the following Equations:

$$D = \frac{R^2T^2}{2n^4F^4C^2\sigma^2}$$

where R is the gas constant, T is the absolute temperature, n is the number of electrons per molecule during oxidization, F is the Faraday constant, C is the concentration of lithium ions, and σ is the Warburg factor, which relates Z_{Re} with the square root of frequency ($\omega^{-1/2}$) in the low-frequency region, as follows:

$$Z_{\text{Re}} = \sigma\omega^{-1/2}$$

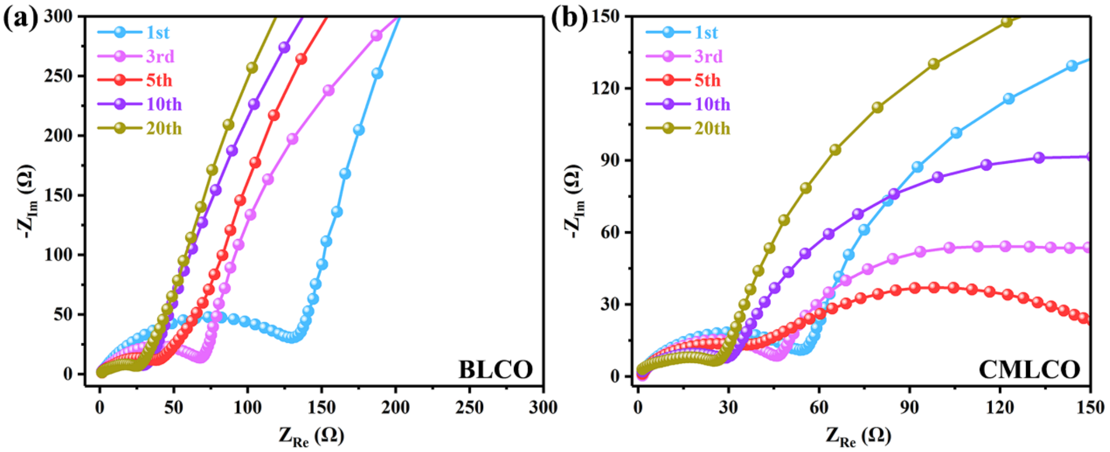

Figure S10 The partially amplified EIS Nyquist plots in Figure 5c,d.

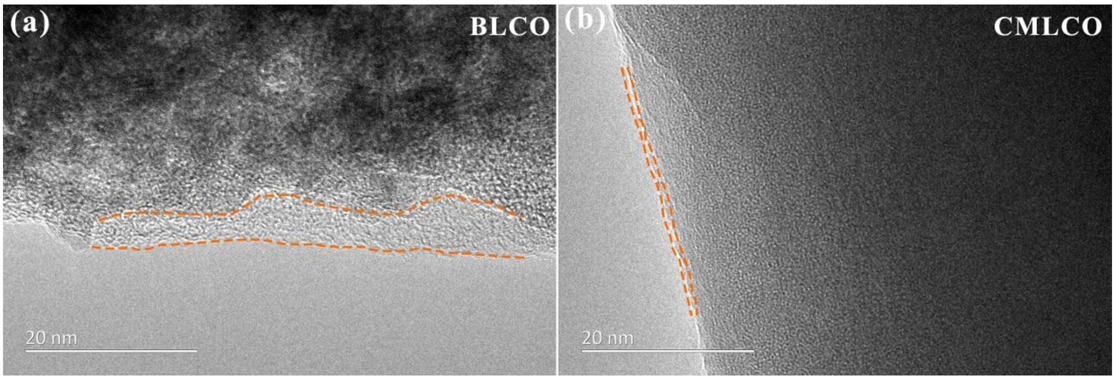

Figure S11 The typical HRTEM images of (a) BLCO and (b) CMLCO cathode after the initial cycle.

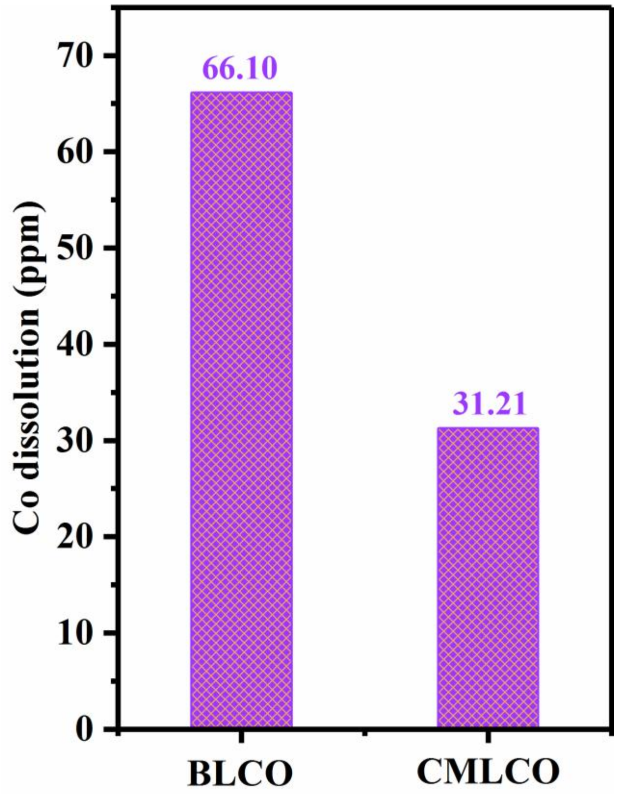

Figure S12 The comparison of Co dissolution in BLCO/Li and CMLCO/Li batteries after 200 cycles.

Table S1 Comparison of electrochemical properties of the reported $LiCoO_2$-based LIBs at the high cutoff voltage of 4.5 V

Modified strategies	1st discharge capacity	Cycling performance	Cell configuration	Ref.
Mg^{2+} doping	184 mAh·g^{-1} at 0.1 C	79% capacity retention after 55 cycles at 1 C	$LiCoO_2$/Li	[4]
Ca^{2+} doping	183 mAh·g^{-1} at 0.1 mA·cm^{-2}	97% capacity retention after 25 cycles at 0.1 mA·cm^{-2}	$LiCoO_2$/Li	[5]
Sn^{2+} doping	162 mAh·g^{-1} at 0.2 C	88% capacity retention after 50 cycles 0.2 C	$LiCoO_2$/Li	[6]
Sr^{2+} doping	184 mAh·g^{-1} at 0.1 C	96% capacity retention after 30 cycles 0.1 C	$LiCoO_2$/Li	[7]
Ba^{2+} doping	170 mAh·g^{-1} at 0.1 mA·cm^{-2}	97% capacity retention after 25 cycles at 0.1 mA·cm^{-2}	$LiCoO_2$/Li	[8]
Al^{3+} doping	155 mAh·g^{-1} at 0.2 C	89% capacity retention after 50 cycles at 0.2 C	$LiCoO_2$/Li	[9]
Ga^{3+} doping	150 mAh·g^{-1} at 55 mA·g^{-1}	67% capacity retention after 30 cycles at 55 mA·g^{-1}	$LiCoO_2$/Li	[10]
Bi^{3+} doping	170 mAh·g^{-1} at 0.2 C	42% capacity retention after 50 cycles at 0.2 C	$LiCoO_2$/Li	[6]
Si^{4+} doping	175 mAh·g^{-1} at 0.4 mA·cm^{-2}	86% capacity retention after 50 cycles at 0.4 mA·cm^{-2}	$LiCoO_2$/Li	[2]
Mn^{2+} doping	180 mAh·g^{-1} at 0.2 C	85% capacity retention after 50 cycles at 0.2 C	$LiCoO_2$/Li	[11]
Cu^{2+} doping	176 mAh·g^{-1} at 0.2 C	74% capacity retention after 50 cycles at 0.2 C	$LiCoO_2$/Li	[11]
Zn^{2+} doping	172 mAh·g^{-1} at 0.2 C	61% capacity retention after 50 cycles at 0.2 C	$LiCoO_2$/Li	[11]
Fe^{2+} doping	160 mAh·g^{-1} at 0.2 C	86% capacity retention after 50 cycles at 0.2 C	$LiCoO_2$/Li	[11]
Cr^{2+} doping	164 mAh·g^{-1} at 0.2 C	66% capacity retention after 50 cycles at 0.2 C	$LiCoO_2$/Li	[6]
Ni^{3+} doping	160 mAh·g^{-1} at 150 mA·g^{-1}	72% capacity retention after 100 cycles at 150 mA·g^{-1}	$LiCoO_2$/Li	[12]
Ti^{4+} doping	165 mAh·g^{-1} at 0.2 C	87% capacity retention after 50 cycles at 0.2 C	$LiCoO_2$/Li	[9]
Zr^{4+} doping	168 mAh·g^{-1} at 0.2 C	87% capacity retention after 50 cycles at 0.2 C	$LiCoO_2$/Li	[6]
Mg^{2+} + Mn^{4+} codoping	146 mAh·g^{-1} at 0.05 C	82% capacity retention after 20 cycles at 0.2 C	$LiCoO_2$/Li	[13]
Al^{3+} + La^{3+} codoping	190 mAh·g^{-1} at 0.1 C	96% capacity retention after 50 cycles at 1/3 C	$LiCoO_2$/Li	[14]
Mg^{2+} + F^{-} codoping	176 mAh·g^{-1} at 32 mA·g^{-1}	89% capacity retention after 50 cycles at 80 mA·g^{-1}	$LiCoO_2$/Li	[15]

Al^{3+} + F^{-} codoping	163 mAh·g^{-1} at 32 mA·g^{-1}	88% capacity retention after 50 cycles at 80 mA·g^{-1}		
Zr^{4+} + F^{-} codoping	160 mAh·g^{-1} at 32 mA·g^{-1}	87% capacity retention after 50 cycles at 80 mA·g^{-1}		
Al_2O_3 coating	172 mAh·g^{-1} at 18 mA·g^{-1}	100% capacity retention after 200 cycles at 500 mA·g^{-1}	$LiCoO_2$/Li	[16]
ZrO_2 coating	168 mAh·g^{-1} at 140 mA·g^{-1}	81% capacity retention after 200 cycles at 0.1 mA·cm^{-2}	$LiCoO_2$/Li	[17]
MgO coating	160 mAh·g^{-1} at 0.1 mA·cm^{-2}	79% capacity retention after 70 cycles at 140 mA·g^{-1}	$LiCoO_2$/Li	[18]
TiO_2 coating	186 mAh·g^{-1} at 140 mA·g^{-1}	86% capacity retention after 100 cycles at 140 mA·g^{-1}	$LiCoO_2$/Li	[19]
ZnO coating	192 mAh·g^{-1} at 0.2 C	96% capacity retention after 50 cycles at 0.2 C	$LiCoO_2$/Li	[20]
SiO_2 coating	170 mAh·g^{-1} at 47 mA·g^{-1}	100% capacity retention after 140 cycles at 47 mA·g^{-1}	$LiCoO_2$/Li	[21]
SnO_2 coating	185 mAh·g^{-1} at 1 C	59% capacity retention after 500 cycles at 1 C	$LiCoO_2$/Li	[22]
La_2O_3 coating	180 mAh·g^{-1} at 54.8 mA·g^{-1}	84% capacity retention after 60 cycles at 54.8 mA·g^{-1}	$LiCoO_2$/Li	[23]
CeO_2 coating	150 mAh·g^{-1} at 0.25 C	77% capacity retention after 50 cycles at 0.25 C	$LiCoO_2$/Li	[24]
CuO coating	165 mAh·g^{-1} at 0.1 C	91% capacity retention after 50 cycles at 0.1 C	$LiCoO_2$/Li	[25]
Fe_2O_3 coating	168 mAh·g^{-1} at 137 mA·g^{-1}	93% capacity retention after 50 cycles at 137 mA·g^{-1}	$LiCoO_2$/Li	[26]
$MgAl_2O_4$ coating	175 mAh·g^{-1} at 16 mA·g^{-1}	96% capacity retention after 70 cycles at 160 mA·g^{-1}	$LiCoO_2$/Li	[27]
$(Y_2O_3)_{0.08}(ZrO_2)_{0.92}$ coating	170 mAh·g^{-1} at 54.8 mA·g^{-1}	96% capacity retention after 35 cycles at 54.8 mA·g^{-1}	$LiCoO_2$/Li	[28]
AlF_3 coating	185 mAh·g^{-1} at 32 mA·g^{-1}	94% capacity retention after 50 cycles at 80 mA·g^{-1}	$LiCoO_2$/Li	[29]
NH_4AlF_4 coating	188.7 mAh·g^{-1} at 36 mA·g^{-1}	85% capacity retention after 50 cycles at 180 mA·g^{-1}	$LiCoO_2$/Li	[30]
MgF_2 coating	180 mAh·g^{-1} at 54.8 mA·g^{-1}	78% capacity retention after 50 cycles at 54.8 mA·g^{-1}	$LiCoO_2$/Li	[31]
LaF_3 coating	169 mAh·g^{-1} at 0.2 mA·cm^{-2}	95% capacity retention after 50 cycles at 0.2 mA·cm^{-2}	$LiCoO_2$/Li	[32]
ZrO_xF_y coating	180 mAh·g^{-1} at 155 mA·g^{-1}	57% capacity retention after 200 cycles at 155 mA·g^{-1}	$LiCoO_2$/Li	[33]
YPO_4 coating	175 mAh·g^{-1} at 0.1 mA·cm^{-2}	90% capacity retention after 50 cycles at 0.1 mA·cm^{-2}	$LiCoO_2$/Li	[34]
$Mg_3(PO_4)_2$ coating	179 mAh·g^{-1} at 18 mA·g^{-1}	75% capacity retention after 50 cycles at 180 mA·g^{-1}	$LiCoO_2$/Li	[35]
$Zn_3(PO_4)_2$ coating	186 mAh·g^{-1} at 18	83% capacity retention after 50		

	$mA·g^{-1}$	cycles at 180 $mA·g^{-1}$		
$MnSiO_4$ coating	180 $mAh·g^{-1}$ at 0.2 $mA·cm^{-2}$	93% capacity retention after 50 cycles at 0.2 $mA·cm^{-2}$	$LiCoO_2$/Li	[36]
$LiNi_{1/3}Co_{1/3}Mn_{1/3}O_2$ coating	183 $mAh·g^{-1}$ at 80 $mA·g^{-1}$	92% capacity retention after 80 cycles at 80 $mA·g^{-1}$	$LiCoO_2$/Li	[37]
$LiCoPO_4$ coating	188 $mAh·g^{-1}$ at 18 $mA·g^{-1}$	81% capacity retention after 50 cycles at 180 $mA·g^{-1}$	$LiCoO_2$/Li	[38]
$Li_{1.2}Mn_{0.6}Ni_{0.2}O_2$ coating	178 $mAh·g^{-1}$ at 0.2 C	81% capacity retention after 100 cycles at 0.2 C	$LiCoO_2$/Li	[39]
$LiAlO_2$ coating	169 $mAh·g^{-1}$ at 0.1 C	96% capacity retention after 20 cycles at 0.1 C	$LiCoO_2$/Li	[40]
Li_3PO_4 coating	185 $mAh·g^{-1}$ at 140 $mA·g^{-1}$	80% capacity retention after 100 cycles at 140 $mA·g^{-1}$	$LiCoO_2$/Li	[41]
Li_3VO_4 coating	162 $mAh·g^{-1}$ at 30 $mA·g^{-1}$	86% capacity retention after 100 cycles at 30 $mA·g^{-1}$	$LiCoO_2$/Li	[42]
$LiMgPO_4$ coating	200 $mAh·g^{-1}$ at at 0.2 $mA·cm^{-2}$	91% capacity retention after 50 cycles at 0.5 $mA·cm^{-2}$	$LiCoO_2$/Li	[43]
LiPON coating	192 $mAh·g^{-1}$ at at 0.2 C	82% capacity retention after 60 cycles at 0.2 C	$LiCoO_2$/Li	[44]
$Li_{1+x}Al_xTi_{2x}(PO_4)_3$ coating	182 $mAh·g^{-1}$ at at 0.2 $mA·cm^{-2}$	89% capacity retention after 60 cycles at 0.5 $mA·cm^{-2}$	$LiCoO_2$/Li	[45]
Li_2CO_3 coating	183 $mAh·g^{-1}$ at 28 $mA·g^{-1}$	88% capacity retention after 50 cycles at 28 $mA·g^{-1}$	$LiCoO_2$/Li	[46]
Li_2ZrO_3 coating	120 $mAh·g^{-1}$ at 1000 $mA·g^{-1}$	83% capacity retention after 100 cycles at 1000 $mA·g^{-1}$	$LiCoO_2$/Li	[47]
$Li_4Ti_5O_{12}$ coating	190 $mAh·g^{-1}$ at 28 $mA·g^{-1}$	90% capacity retention after 60 cycles at 28 $mA·g^{-1}$	$LiCoO_2$/Li	[48]
PI/PVP coating	182 $mAh·g^{-1}$ at 0.5 C	74% capacity retention after 50 cycles at 0.5 C	$LiCoO_2$/Li	[49]
Superficial P doping (Li–O–P glassphase)	198 $mAh·g^{-1}$ at 0.1 C	55% capacity retention after 100 cycles at 0.1 C	$LiCoO_2$/Li	[50]
C60 coating	175 $mAh·g^{-1}$ at 0.1 C	69% capacity retention after 50 cycles at 0.1 C	$LiCoO_2$/Li	[51]
PAN coating	182 $mAh·g^{-1}$ at 0.2 C	95% capacity retention after 60 cycles at 0.7 C	$LiCoO_2$/Li	[52]
PPy coating	182 $mAh·g^{-1}$ at 75 $mA·g^{-1}$	95% capacity retention after 170 cycles at 75 $mA·g^{-1}$	$LiCoO_2$/Li	[53]
Al-doped ZnO coating	185 $mAh·g^{-1}$ at 20 $mA·g^{-1}$	95% capacity retention after 200 cycles at 100 $mA·g^{-1}$	$LiCoO_2$/Li	[54]
$LiAlPO_{3.93}F_{1.07}$ coating	206	92% capacity retention after 50 cycles at 100 $mA·g^{-1}$	$LiCoO_2$/Li	[55]
Mg doping + $Li_4Ti_5O_{12}$ coating	185 $mAh·g^{-1}$ at 160 $mA·g^{-1}$	76% capacity retention after 50 cycles at 160 $mA·g^{-1}$	$LiCoO_2$/Li	[56]
Mg doping +	178 $mAh·g^{-1}$ at 160	57% capacity retention after 50		

Li_2TiO_3 coating	$mA\cdot g^{-1}$	cycles at 160 $mA\cdot g^{-1}$		
Mg doping + $Li_{1.3}Al_{0.3}Ti_{1.7}(PO_4)_3$ coating	185 $mAh\cdot g^{-1}$ at 1 C	96% capacity retention after 100 cycles at 1C	$LiCoO_2$/Li	[57]
Mg doping + ZrO_xF_y coating	167 $mAh\cdot g^{-1}$ at 15.5 $mA\cdot g^{-1}$	91% capacity retention after 100 cycles at 155 $mA\cdot g^{-1}$	$LiCoO_2$/Li	[58]
Al+Ti bulk codoping + gradient surface Mg doping	212 $mAh\cdot g^{-1}$ at 14 $mA\cdot g^{-1}$	77% (93%, 88%) capacity retention after 200 (50, 100) cycles at 140 $mA\cdot g^{-1}$	$LiCoO_2$/Li	This work

Table S2 Comparison of electrochemical properties of the reported $LiCoO_2$-based LIBs at the high cutoff voltage of 4.6 V

Modified strategies	1st discharge capacity	Cycling performance	Cell configuration	Ref.
W doping	223 $mAh{\cdot}g^{-1}$ at 27.4 $mA{\cdot}g^{-1}$	72.3% capacity retention after 100 cycles at 274 $mA{\cdot}g^{-1}$	$LiCoO_2$/Li	[59]
Mg+Cu codoping	208 $mAh{\cdot}g^{-1}$ at 0.2 C	88% capacity retention after 100 cycles at 0.2 C	$LiCoO_2$/Li	[60]
Al_2O_3 coating	210 $mAh{\cdot}g^{-1}$ at 14 $mA{\cdot}g^{-1}$	70% capacity retention after 50 cycles at 140 $mA{\cdot}g^{-1}$	$LiCoO_2$/Li	[61]
ZrO_2 coating	217 $mAh{\cdot}g^{-1}$ at 220 $mA{\cdot}g^{-1}$	55% capacity retention after 200 cycles at 220 $mA{\cdot}g^{-1}$	$LiCoO_2$/Li	[62]
La_2O_3 coating	194 $mAh{\cdot}g^{-1}$ at 54.8 $mA{\cdot}g^{-1}$	83% capacity retention after 60 cycles at 54.8 $mA{\cdot}g^{-1}$	$LiCoO_2$/Li	[23]
Co_3O_4 coating	164 $mAh{\cdot}g^{-1}$ at 150 $mA{\cdot}g^{-1}$	62% capacity retention after 50 cycles at 150 $mA{\cdot}g^{-1}$	$LiCoO_2$/Li	[63]
$(Y_2O_3)_{0.08}(ZrO_2)_{0.92}$ coating	207 $mAh{\cdot}g^{-1}$ at 54.8 $mA{\cdot}g^{-1}$	78% capacity retention after 35 cycles at 54.8 $mA{\cdot}g^{-1}$	$LiCoO_2$/Li	[28]
AlF_3 coating	225 $mAh{\cdot}g^{-1}$ at 32 $mA{\cdot}g^{-1}$	80% capacity retention after 35 cycles at 80 $mA{\cdot}g^{-1}$	$LiCoO_2$/Li	[29]
CeF_3 coating	170 $mAh{\cdot}g^{-1}$ at 40 $mA{\cdot}g^{-1}$	94% capacity retention after 20 cycles at 40 $mA{\cdot}g^{-1}$	$LiCoO_2$/Li	[64]
ZrO_xF_y coating	215 $mAh{\cdot}g^{-1}$ at 15.5 $mA{\cdot}g^{-1}$	33% capacity retention after 200 cycles at 155 $mA{\cdot}g^{-1}$	$LiCoO_2$/Li	[33]
$AlPO_4$ coating	210 $mAh{\cdot}g^{-1}$ at 140 $mA{\cdot}g^{-1}$	76% capacity retention after 50 cycles at 140 $mA{\cdot}g^{-1}$	$LiCoO_2$/Li	[65]
$FePO_4$ coating	212 $mAh{\cdot}g^{-1}$ at 140 $mA{\cdot}g^{-1}$	50% capacity retention after 50 cycles at 140 $mA{\cdot}g^{-1}$		
$CePO_4$ coating	216 $mAh{\cdot}g^{-1}$ at 140 $mA{\cdot}g^{-1}$	19% capacity retention after 50 cycles at 140 $mA{\cdot}g^{-1}$		
$SrPO_4$ coating	214 $mAh{\cdot}g^{-1}$ at 140 $mA{\cdot}g^{-1}$	39% capacity retention after 50 cycles at 140 $mA{\cdot}g^{-1}$		
$LiAlO_2$ coating	230 $mAh{\cdot}g^{-1}$ at 10 $mA{\cdot}g^{-1}$	82% capacity retention after 50 cycles at 50 $mA{\cdot}g^{-1}$	$LiCoO_2$/Li	[66]
Li_2CO_3 coating	192 $mAh{\cdot}g^{-1}$ at 28 $mA{\cdot}g^{-1}$	70% capacity retention after 50 cycles at 28 $mA{\cdot}g^{-1}$	$LiCoO_2$/Li	[46]
PI coating	200 $mAh{\cdot}g^{-1}$ at 0.5C	64% capacity retention after 50 cycles at 0.5C	$LiCoO_2$/Li	[67]
Mg doping + ZrO_xF_y coating	200 $mAh{\cdot}g^{-1}$ at 15.5 $mA{\cdot}g^{-1}$	77% capacity retention after 100 cycles at 155 $mA{\cdot}g^{-1}$	$LiCoO_2$/Li	[68]
Li-Al-F-based subsurface doping and surface coating	208 $mAh{\cdot}g^{-1}$ at 27.4 $mA{\cdot}g^{-1}$	81.8% (89.1%) capacity retention after 200 (100) cycles at 27.4 $mA{\cdot}g^{-1}$	$LiCoO_2$/Li	[69]
Al+Ti bulk	225 $mAh{\cdot}g^{-1}$ at 14	78% (86%) capacity retention	$LiCoO_2$/Li	This

codoping + gradient surface	$mA \cdot g^{-1}$	after 200 (100) cycles at 70 $mA \cdot g^{-1}$		work
Mg doping	208 $mAh \cdot g^{-1}$ at 14 $mA \cdot g^{-1}$	78% (85%) capacity retention after 200 (100) cycles at 70 $mA \cdot g^{-1}$	$LiCoO_2$/MCMB	
	200 $mAh \cdot g^{-1}$ at 70 $mA \cdot g^{-1}$	27% (55%, 40%) capacity retention after 200 (50, 100) cycles at 70 $mA \cdot g^{-1}$	$LiCoO_2$/MCMB (60 ºC)	

Table S3 The variation in the resistances of BLCO and CMLCO charged up to 4.2, 4.5 and 4.6 V at first cycle

Cathode	Voltage (V)	R_e (Ω)	R_{sf} (Ω)	R_{ct} (Ω)
BLCO	4.2	0.9780	68.55	32.62
	4.5	1.028	57.75	80.55
	4.6	1.056	70.05	542.2
CMLCO	4.2	0.6774	22.65	38.27
	4.5	0.6091	31.67	46.74
	4.6	0.8307	32.75	133.05

Table S4 The variation in the resistances of BLCO and CMLCO fully discharged at 1st, 3rd, 5th, 10th and 20th cycles

Cathode	Cycle	R_e (Ω)	R_{sf} (Ω)	R_{ct} (Ω)
BLCO	1st	0.9548	138.5	2262
	3rd	0.6838	73.18	923.8
	5th	0.6023	35.38	1302

	10th	0.5084	32.16	1687
	20th	0.5143	28.62	2060
CMLCO	1st	0.9812	51.16	236.72
	3rd	0.9322	43.62	72.28
	5th	0.8450	34.43	126.4
	10th	0.7036	30.98	214.8
	20th	0.6449	29.89	416.2

References

[1] S. Venkatraman, V. Subramanian, S. G. Kumar, N. G. Renganathan, N. Muniyandi, *Electrochem. Commun.* **2000**, 2, 18.

[2] Y. Jin, P. Lin, C. H. Chen, *Solid State Ionics* **2006**, 177, 317.

[3] a) T. Chen, X. Li, H. Wang, X. Yan, L. Wang, B. Deng, W. Ge, M. Qu, *J. Power Sources* **2018**, 374, 1; b) R. P. Qing, J. L. Shi, D. D. Xiao, X. D. Zhang, Y. X. Yin, Y. B. Zhai, L. Gu, Y. G. Guo, *Adv. Energy Mater.* **2016**, 6; c) C. L. Xu, W. Xiang, Z. G. Wu, Y. D. Xu, Y. C. Li, M. Z. Chen, G. XiaoDong, G. P. Lv, J. Zhang, B. H. Zhong, *ACS Appl. Mater. Interfaces* **2018**, 10, 27821.

[4] R. Z. Yin, Y. S. Kim, S. J. Shin, I. Jung, J. S. Kim, S. K. Jeong, *J. Electrochem. Soc.* **2012**, 159, A253.

[5] R. Sathiyamoorthi, P. Shakkthivel, T. Vasudevan, *Mater. Lett.* **2007**, 61, 3746.

[6] M. J. Zou, M. Yashio, S. Gopukumar, J. I. Yamaki, *Chem. Mater.* **2005**, 17, 1284.

[7] S. Valanarasu, R. Chandramohan, *J. Alloy. Compd.* **2010**, 494, 434.

[8] R. Sathiyamoorthi, P. Shakkthivel, R. Gangadharan, T. Vasudevan, *Mater. Chem. Phys.* **2007**, 104, 403.

[9] M. J. Zou, M. Yoshio, S. Gopukumar, J. Yamaki, *Electrochem. Solid St.* **2004**, 7, A176.

[10] S. M. Lala, L. A. Montoro, V. Lemos, M. Abbate, J. M. Rosolen, *Electrochim. Acta* **2005**, 51, 7.

[11] M. J. Zou, M. Yoshio, S. Gopukumar, J. Yamaki, *Chem. Mater.* **2003**, 15, 4699.

[12] J. Liang, D. H. Wu, M. Hu, Y. Tian, J. P. Wei, Z. Zhou, *Electrochim. Acta* **2014**, 146, 784.

[13] W. B. Luo, X. H. Li, J. R. Dahn, *J. Electrochem. Soc.* **2010**, 157, A993.

[14] Q. Liu, X. Su, D. Lei, Y. Qin, J. G. Wen, F. M. Guo, Y. M. A. Wu, Y. C. Rong, R. H. Kou, X. H. Xiao, F. Aguesse, J. Bareno, Y. Ren, W. Q. Lu, Y. X. Li, *Nat. Energy* **2018**, 3, 936.

[15] H. G. Jung, N. V. Gopal, J. Prakash, D. W. Kim, Y. K. Sun, *Electrochim. Acta* **2012**, 68, 153.

[16] I. D. Scott, Y. S. Jung, A. S. Cavanagh, Y. F. An, A. C. Dillon, S. M. George, S. H. Lee, *Nano Lett.* **2011**, 11, 414.

[17] X. F. Li, J. Liu, X. B. Meng, Y. J. Tang, M. N. Banis, J. L. Yang, Y. H. Hu, R. Y. Li, M. Cai, X. L. Sun, *J. Power Sources* **2014**, 247, 57.

[18] Z. X. Wang, C. A. Wu, L. J. Liu, F. Wu, L. Q. Chen, X. J. Huang, *J. Electrochem. Soc.* **2002**, 149, A466.

[19] A. Zhou, Y. Lu, Q. Wang, J. Xu, W. Wang, X. Dai, J. Li, *J. Power Sources* **2017**, 346, 24.

[20] X. Dai, L. Wang, J. Xu, Y. Wang, A. Zhou, J. Li, *ACS Appl. Mater. Interfaces* **2014**, 6, 15853.

[21] Z. H. Chen, J. R. Dahn, *Electrochim. Acta* **2004**, 49, 1079.

[22] C. Hudaya, J. H. Park, J. K. Lee, W. Choi, *Solid State Ionics* **2014**, 256, 89.

[23] G. T. K. Fey, P. Muralidharan, C. Z. Lu, Y. D. Cho, *Electrochim. Acta* **2006**, 51, 4850.

[24] H. W. Ha, N. J. Yun, M. H. Kim, M. H. Woo, K. Kim, *Electrochim. Acta* **2006**, 51, 3297.

[25] Q. Hao, H. Y. Ma, Z. C. Ju, G. D. Li, X. W. Li, L. Q. Xu, Y. T. Qian, *Electrochim. Acta* **2011**, 56, 9027.

[26] Q. Hao, C. X. Xu, S. Z. Jia, X. Y. Zhao, *Electrochim. Acta* **2013**, 113, 439.

[27] D. D. Liang, H. F. Xiang, X. Liang, S. Cheng, C. H. Chen, *RSC Adv.* **2017**, 7, 6809.

[28] G. T. K. Fey, C. L. Hsiao, P. Muralidharan, *J. Power Sources* **2009**, 189, 837.

[29] Y. K. Sun, C. S. Yoon, S. T. Myung, I. Belharouak, K. Amine, *J. Electrochem. Soc.* **2009**, 156, A1005.

[30] R. Amine, H. H. Sun, H. J. Sun, J. Prakash, *Electrochem. Solid St.* **2010**, 13, A101.

[31] Y. Bai, K. Jiang, S. W. Sun, Q. Wu, X. Lu, N. Wan, *Electrochim. Acta* **2014**, 134, 347.

[32] Z. Yang, Q. Qiao, W. Yang, *Electrochim. Acta* **2011**, 56, 4791.

[33] Z. G. Wang, Z. X. Wang, H. J. Guo, W. J. Peng, X. H. Li, J. X. Wang, *Mater. Lett.* **2014**, 123, 93.

[34] Y. Bai, Y. F. Yin, N. Liu, B. K. Guo, H. Shi, J. Y. Liu, Z. X. Wang, L. Q. Chen, *J. Power Sources* **2007**, 174, 328.

[35] J. Eom, J. Cho, *J. Electrochem. Soc.* **2008**, 155, A201.

[36] Z. X. Yang, W. S. Yang, D. G. Evans, G. Li, Y. Y. Zhao, *Electrochem. Commun.* **2008**, 10, 1136.

[37] S. M. Park, T. H. Cho, Y. M. Kim, M. Yoshio, *Electrochem. Solid St.* **2005**, 8, A299.

[38] H. Lee, M. G. Kim, J. Cho, *Electrochem. Commun.* **2007**, 9, 149.

[39] G. R. Hu, J. C. Cao, Z. D. Peng, Y. B. Cao, K. Du, *Electrochim. Acta* **2014**, 149, 49.

[40] H. Cao, B. J. Xia, Y. Zhang, N. X. Xu, *Solid State Ionics* **2005**, 176, 911.

[41] A. J. Zhou, J. Xu, X. Y. Dai, B. Yang, Y. T. Lu, L. P. Wang, C. Fan, J. Z. Li, *J. Power Sources* **2016**, 322, 10.

[42] X. Pa, C. H. Yu, *Nanoscale* **2012**, 4, 6743.

[43] H. Morimoto, H. Awano, J. Terashima, S. Nakanishi, Y. Hirama, K. Ishikawa, S.-i. Tobishima, *J. Power Sources* **2012**, 211, 66.

[44] K. H. Choi, J. H. Jeon, H. K. Park, S. M. Lee, *J. Power Sources* **2010**, 195, 8317.

[45] H. Morimoto, H. Awano, J. Terashima, Y. Shindo, S. Nakanishi, N. Ito, K. Ishikawa, S. Tobishima, *J. Power Sources* **2013**, 240, 636.

[46] X. Y. Dai, A. J. Zhou, J. Xu, Y. T. Lu, L. P. Wang, C. Fan, J. Z. Li, *J. Phys. Chem. C* **2016**, 120, 422.

[47] J. C. Zhang, R. Gao, L. M. Sun, H. Zhang, Z. B. Hu, X. F. Liu, *Electrochim. Acta* **2016**, 209, 102.

[48] A. J. Zhou, X. Y. Dai, Y. T. Lu, Q. J. Wang, M. S. Fu, J. Z. Li, *ACS Appl. Mater. Interfaces* **2016**, 8, 34123.

[49] J. M. Kim, J. H. Park, C. K. Lee, S. Y. Lee, *Sci. Rep.* **2014**, 4, 4602.

[50] S. H. Min, M. R. Jo, S. Y. Choi, Y. I. Kim, Y. M. Kang, *Adv. Energy Mater.* **2016**, 6, 1501717.

[51] C. Hudaya, M. Halim, J. Proll, H. Besser, W. Choi, W. Pfleging, H. J. Seifert, J. K. Lee, *J. Power Sources* **2015**, 298, 1.

[52] X. H. Yang, L. Y. Shen, B. Wu, Z. C. Zuo, D. B. Mu, B. R. Wu, H. H. Zhou, *J. Alloy Compd.* **2015**, 639, 458.

[53] J. C. Cao, G. R. Hu, Z. D. Peng, K. Du, Y. B. Cao, *J. Power Sources* **2015**, 281, 49.

[54] B. Shen, P. J. Zuo, Q. Li, X. S. He, G. P. Yin, Y. L. Ma, X. Q. Cheng, C. Y. Du, Y. Z. Gao, *Electrochim. Acta* **2017**, 224, 96.

[55] B. Shen, Q. Liu, L. Wang, G. Yin, P. Zuo, Y. Ma, X. Cheng, C. Du, Y. Gao, *Electrochem. Commun.* **2017**, 83, 106.

[56] J. H. Shim, J. Lee, S. Y. Han, S. Lee, *Electrochim. Acta* **2015**, 186, 201.

[57] J. H. Shim, J. M. Han, J. H. Lee, S. Lee, *ACS Appl. Mater. Interfaces* **2016**, 8, 12205.

[58] Z. G. Wang, Z. X. Wang, H. J. Guo, W. J. Peng, X. H. Li, *Ceram. Int.* **2015**, 41, 469.

[59] J. N. Zhang, Q. H. Li, Q. Li, X. Q. Yu, H. Li, *Chinese Phys. B* **2018**, 27.

[60] C. Nithya, R. Thirunakaran, A. Sivashanmugam, S. Gopukumar, *ACS Appl. Mater. Interfaces* **2012**, 4, 4040.

[61] J. Cho, T. G. Kim, C. Kim, J. G. Lee, Y. W. Kim, B. Park, *J. Power Sources* **2005**, 146, 58.

[62] H. Miyashiro, A. Yamanaka, M. Tabuchi, S. Seki, M. Nakayama, Y. Ohno, Y. Kobayashi, Y. Mita, A. Usami, M. Wakihara, *J. Electrochem. Soc.* **2006**, 153, A348.

[63] S. Jeong, S. Park, J. Cho, *Adv. Energy Mater.* **2011**, 1, 368.

[64] A. Aboulaich, K. Ouzaouit, H. Faqir, A. Kaddami, I. Benzakour, I. Akalay, *Mater. Res. Bull.* **2016**, 73, 362.

[65] J. G. Lee, T. G. Kim, B. Park, *Mater. Res. Bull.* **2007**, 42, 1201.

[66] J. Xie, J. Zhao, Y. Y. Liu, H. T. Wang, C. Liu, T. Wu, P. C. Hsu, D. C. Lin, Y. Jin, Y. Cui, *Nano Res.* **2017**, 10, 3754.

[67] J. H. Park, J. H. Cho, J. S. Kim, E. G. Shim, S. Y. Lee, *Electrochim. Acta* **2012**, 86, 346.

[68] Z. G. Wang, Z. X. Wang, H. J. Guo, W. J. Peng, X. H. Li, G. C. Yan, J. X. Wang, *J. Alloy Compd.* **2015**, 621, 212.

[69] J. Qian, L. Liu, J. Yang, S. Li, X. Wang, H. L. Zhuang, Y. Lu, *Nat. Commun.* **2018**, 9, 4918.
